# Diagnosis and Non-Invasive Treatment of Obesity in Adults with Type 2 Diabetes Mellitus: A Review of Guidelines

**DOI:** 10.3390/jcm12134431

**Published:** 2023-06-30

**Authors:** Michał Łuniewski, Beata Matyjaszek-Matuszek, Monika Lenart-Lipińska

**Affiliations:** Department of Endocrinology, Diabetology and Metabolic Diseases, Medical University of Lublin, 20-954 Lublin, Poland; beata.matyjaszek-matuszek@umlub.pl (B.M.-M.); monika.lenart-lipinska@umlub.pl (M.L.-L.)

**Keywords:** weight management, medical nutrition therapy, GLP1-RA, SLGT2 inhibitor

## Abstract

Obesity, a chronic disease with multifactorial etiopathogenesis, is characterized by excessive accumulation of adipose tissue. Obesity prevalence is growing globally at an alarming rate. The overwhelming majority of obesity cases are caused by inappropriate lifestyles, such as overconsumption of food and inadequate physical activity. Metabolic and biochemical changes due to increased adiposity resulted in numerous comorbidities, increased all-cause mortality, and reduced quality of life. T2DM (type 2 diabetes mellitus) and obesity have many common pathogenetic points and drive each other in a vicious cycle. The aim of this article is to review obesity management guidelines and highlight the most important points. Management of both obesity-related and T2DM complications incur enormous expenses on healthcare systems. It is, therefore, paramount to provide streamlined yet custom-tailored weight management in order to avoid the negative ramifications of both diseases. Efficient obesity treatment leads to better diabetes control since some antidiabetic medications support weight reduction. Obesity treatment should be overseen by a multi-disciplinary team providing indispensable information and individually tailored regimens to patients. Weight management should be multimodal and consist chiefly of MNT (medical nutrition therapy), physical activity, and lifestyle changes. A comprehensive approach to obesity treatment may give tangible results to quality of life and comorbidities.

## 1. Introduction

Obesity is a chronic disease characterized by an overaccumulation of adipose tissue and by complex etiopathogenesis. The overwhelming majority of obesity cases are caused by inappropriate lifestyles, such as excessive energy consumption and inadequate physical activity [1]. Other important factors that significantly increase the risk of obesity include comorbidities and unrestricted access to palatable food, as well as genetic, social, cultural, mental, iatrogenic factors (e.g., medications), and some diseases (e.g., endocrine disorders) [2]. Metabolic and biochemical changes due to a disproportionately large percentage of adipose tissue in the total body weight result in an increased incidence of complications affecting most organ systems, especially the CVS (cardiovascular system) such as an increased risk of myocardial infarction, stroke, and all-cause cardiovascular mortality [3].

Obesity is most commonly defined as a BMI (body mass index) ≥ 30 kg/m^2^, while overweight is defined as a BMI ≥ 25 kg/m^2^ [3,4]. The Asian population has a greater propensity to accumulate central adipose tissue compared to other populations and has higher mortality from obesity-caused diseases and complications. Therefore, different BMI cut-off values were adopted for the Asian population, e.g., the WHO expert consultation, the Asia-Pacific classification of BMI, and the Indian Consensus Group for Asian Indians [5,6,7,8,9]. Some researchers advocate the use of alternative obesity definitions, especially for abdominal obesity, e.g., definitions based on waist circumference, visceral fat mass, or fat percentage (fat percentage ≥ 25% for women and ≥30% for men) [10]. BMI is often insufficient to assess the cardiometabolic risk of an obese patient; therefore, Canadian authors recommend waist circumference measurement as a direct predictor of the amount of pericardial fat and visceral fat accumulated in the abdominal cavity between organs. For a more precise clinical assessment, obesity has been further subdivided into (Table 1) overweight, obesity class I, obesity class II, and obesity class III [4,11]. While BMI provides a quick and simple diagnosis, it has its own shortcomings and, thus, is not an ideal tool (e.g., in bodybuilders or people with sarcopenia).

Due to the social changes taking place in the twentieth century and the gradual yet steady spread of improper eating habits, obesity became so prevalent that in 1997 that it was the first civilization, non-infectious disease formally recognized by the WHO (World Health Organization) as a “global epidemic” [12]. The AMA (American Medical Association) categorized obesity as a disease in 2013 [13].

## 2. The Aim of the Review

Available guidelines demonstrate that national/international recommendations on obesity treatment significantly differ from one another. The purpose of this review is to identify the most important common points and find the factors that set different guidelines apart. Moreover, the aim of the review is to highlight key steps in obesity management in patients with T2DM (type 2 diabetes mellitus), so that the diagnostic and therapeutic process is streamlined and efficient.

The subsequent main body of the text is based principally on the guidelines put forth by the ADA (American Diabetes Association), DC (Diabetes Canada), and the EASD (European Association for the Study of Diabetes), with supplementary information based on various studies by other research teams, authors, etc. [14,15,16,17,18,19,20,21].

## 3. The Significance of the Interrelationship between Obesity and T2DM

Multi-year epidemiological observations prove that people with obesity develop T2DM more readily [2,22]. Vice versa, people with T2DM have a stronger propensity for obesity compared to the general population. In fact, even values in the upper range or normal BMI values (BMI > 22 kg/m^2^) are considered to carry an increased risk of developing T2DM [23].

**Figure 1:**
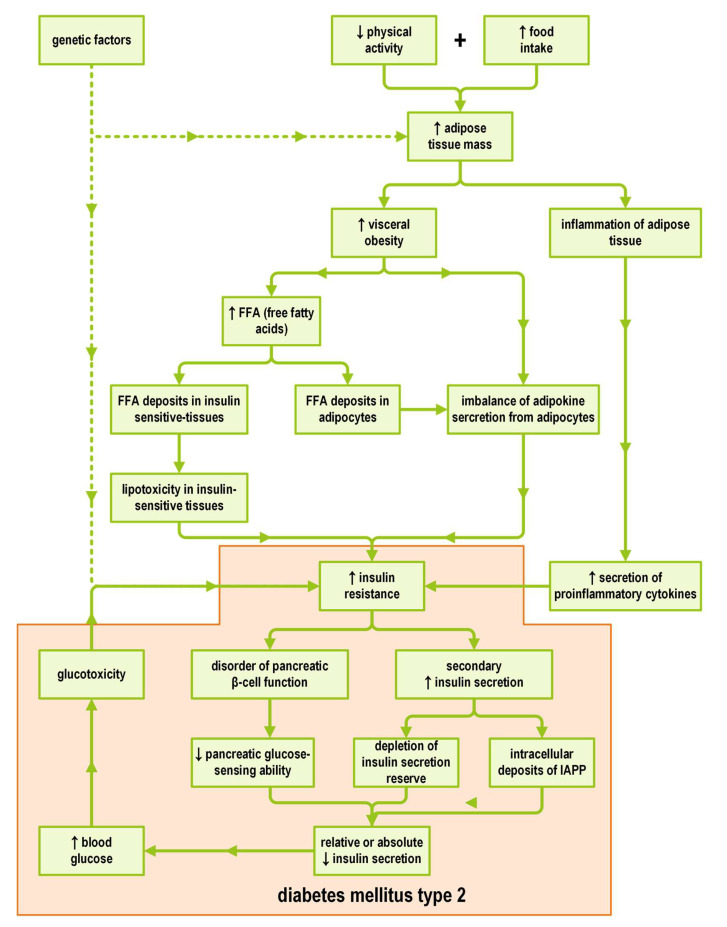
The vicious cycle of obesity, insulin resistance, and diabetes type 2. IAPP (islet amyloid polypeptide). ↑ = an increase, ↓ = a decrease.

Studies have identified pathogenetic processes that contribute to the vicious cycle of obesity and T2DM driving each other (Figure 1) [24].

Obesity causes insulin resistance, which in the long-term turns into prediabetes, and then T2DM. Insulin resistance in obesity is caused by numerous factors including but not limited to the excess of (1) FFAs (free fatty acids) and (2) pro-inflammatory substances, which are both secreted by adipose tissue, as well as (3) abnormal secretion of adipocytokines [25].

The excess of FFAs reduces glucose uptake by the liver and muscle tissue. The FFAs themselves are converted into TGs (triglycerides) and then stored in adipocytes, impairing both their metabolism and secretory functions [26]. TGs—much like glucose—are a source of energy in the process of aerobic respiration. A significant amount of ROS (reactive oxygen species) is generated during the aerobic metabolism of TGs. Since ROS causes damage to cellular structures, cells activate defense mechanisms to protect themselves from excess energy influx and, thus, from further cellular damage. Cells down-regulate both the density of insulin receptors and the number of membrane glucose transporter 4 (GLUT4) molecules [27]. This down-regulation results in a diminished cellular response to insulin.

As emphasized by Mahgoub et al., insulin resistance has a much broader and multifactorial background [28]. Not only obesity but also smoking, inadequate physical activity, and excessive consumption of alcoholic beverages are key factors contributing to insulin resistance [28]. All these processes affect the ability of insulin-dependent tissues, mainly the liver, adipose tissue, and skeletal muscle, to metabolize glucose. The paramount postulated mechanism of the development of insulin resistance is the reduced phosphorylation response of IRS-1 and IRS-2 (insulin receptor substrate-1 and -2) [28,29,30,31]. This, in turn, leads to further down-regulation of on-cell membranes in insulin-dependent tissues, followed by reduced glucose uptake by these tissues and subsequent hyperglycemia.

Mahgoub et al. report that another postulated mechanism leading to insulin resistance is lipotoxicity resulting from the storage of excessive FFAs in the liver, pancreas, and muscles [28,32,33].

Adipose tissue does not remain metabolically or endocrinologically inert. It secretes a number of hormonally active substances, called adipokines, which regulate—in a broad sense—the body’s energy metabolism. Among such substances, adiponectin, leptin, resistin, and TNF-α (tumor necrosis factor α) can be enumerated [28,34,35,36]. In obesity, there is an imbalance in the proportions of secreted adipokines, favoring the overproduction of adipokines that promote the onset of insulin resistance and advance obesity development. TNF-α secretion is increased, whereas adiponectin and leptin secretion is decreased [28,37,38]. Low-grade inflammation in obesity leads to a further oversecretion of TNF-α, a proinflammatory cytokine, but it also holds true for other proinflammatory cytokines, including IL-1 (interleukin 1), IL-6 (interleukin 6), NF-κB (nuclear factor kappa B), and CRP (C-reactive protein), which are secreted in excess and which, by acting synergistically with the abnormal adipokines levels, promote insulin resistance [28,39].

In addition, insulin resistance in obese individuals results from improperly low secretion and increased degradation of incretin hormones, such as GLP-1 (glucagon-like peptide 1) and GIP (gastric inhibitory polypeptide) [40,41]. Incretin hormones are responsible for enhancing the action of insulin, and, therefore, their deficiency leads to increased insulin resistance. In the event of rising insulin resistance, in order to maintain euglycemia, β cells of pancreatic islets attempt to compensate for insulin resistance by increasing insulin secretion. Hyperinsulinemia occurs not only in the postprandial period but also in the fasting period. Insulin resistance, hyperinsulinemia, and preserved normal blood glucose levels (both fasting and postprandial) can last for years. With the passing of time, however, as insulin resistance exacerbates and pancreatic insulin reserve depletes, insulin secretion is no longer sufficient to ensure carbohydrate homeostasis [42]. Long-term hyperglycemia impairs the ability of pancreatic β-cells to secrete insulin by, e.g., the dysregulation of cellular processes that determine glucose-dependent insulin secretion or by intracellular deposition of substances co-secreted with insulin, such as IAPP (islet amyloid polypeptide) [28,43,44]. The subsequent depletion of β-cell insulin secretory capacity results in the clinical manifestation of insulin resistance, manifesting from both relative but also absolute insulin deficiency that develops in long-standing T2DM. When overviewing the natural history of T2DM, the insulin resistance transforms primarily into prediabetes and then into overt T2DM when insulin secretion is insufficient to maintain glucose levels within the normal range.

Undoubtedly, as evidenced in earlier paragraphs, the development of insulin resistance has a multifactorial background. A crucial role is played by (not yet fully understood) genetic factors or modifiable factors, e.g., inadequate physical activity. The latter affects, among other things, the glucose uptake capacity of insulin-dependent tissues, especially muscles, through the down-regulation of GLUT-4 [28,45].

Clearly, some of the causes of insulin resistance are directly due to obesity itself or due to obesity sequelae, while some are independent of obesity but consequently result in the exacerbation of obesity. T2DM—even in patients within the normal BMI range—manifests itself as hyperinsulinemia. An excess of insulin, a hormone with orexigenic and anabolic effects, leads to appetite stimulation, weight gain and, consequently, obesity [46].

## 4. Obesity Diagnosis and Treatment Plan

Before initiation of the treatment, obesity should be diagnosed, at least in a basic scope, and preferably comprehensively. The patient’s height and weight should be measured, and BMI calculated. The degree of obesity needs to be classified based on BMI value (Table 1). BMI calculation should be performed not only at therapy commencement but also periodically throughout the duration of therapy (at least yearly) [47]. The background of obesity (cultural, environmental, genetic factors, somatic diseases, etc.) should be investigated by obtaining a thorough medical history from the patient [48]. If suspicion of a somatic disease as an obesity cause arises (e.g., an endocrine disorder, chromosomal/genetic defect, etc.), an inpatient work-up should be considered. Treatment of the root cause, whenever possible, is preferred [21].

The patient should be informed about possible obesity-related complications (premature all-cause mortality risk and increased morbidity, especially from cardiovascular causes) and encouraged to lose weight in order to avoid these negative outcomes [21,49]. The patient’s questions should be meticulously answered, and any doubts dispelled. The physician and the patient should develop a joint treatment plan, taking into account the patient’s motivation, preferences regarding weight loss methods, and weight reduction goals [50]. Some treatment options should be recommended to every patient regardless of accompanying factors. These include dietary treatment and physical exercise [51,52]. Pharmacotherapy and bariatric surgery are reserved for strictly defined groups of patients and are complementary to basic treatment options.

Diet and physical exercise can achieve ≥5% weight loss from the baseline. At the very least, a weight reduction of 3–5% from the baseline is required for apparent health benefits (the greater the weight loss, the greater the benefits) [47,53].

Apart from weight reduction, there are also numerous other advantages of lifestyle change, such as a decrease in fasting and postprandial glycemia, a decrease in HbA1c, TGs, and blood pressure, as well as an increase in HDL [54,55]. All of these translate into better diabetes control and a reduction in overall cardiovascular mortality [56,57]. In some cases, lifestyle changes can achieve not only a reduction in antidiabetic medications dosage, but even their complete discontinuation [58]. This is within reason, especially in the first six years after a diabetes diagnosis had been made [19]. The AHEAD (Action for Health in Diabetes) trial proved other merits of weight loss, including improvement in sexual and motor functions and improvement in QoL (quality of life) [59,60].

The effectiveness of therapy is greater if supervised by an interdisciplinary team of various specialists (diabetologists experienced in obesity management, primary care physicians, registered dietitians, diabetes counselors, etc.) compared to interventions undertaken by physicians alone. [44] Therapeutic meetings with healthcare providers should be frequent: ≥16 sessions over 6 months (ADA) or 12–26 sessions over 6–12 months (EASD) [14,19].

## 5. Medical Nutrition Therapy (MNT) for Obesity Treatment in T2DM

Dietary treatment plays a key role in the treatment of obesity regardless of its root cause and comorbidities (e.g., T2DM) [21,51]. MNT includes not only diet planning and the entire process of creating and retaining proper eating habits, but also ongoing nutritional education so that the patient can make appropriate dietary choices in the future.

The ADA recommends an overall energy deficit of 500–750 kcal/day. This roughly translates into a daily caloric content in the diet equal to 1200–1500 kcal/day for women and 1500–1800 kcal/day for men. “Joslin Clinical Nutrition Guideline for Overweight and Obese Adults With Type 2 Diabetes (T2D) or Prediabetes, or Those at High Risk for Developing T2D” suggests that the caloric deficit in the diet should be 250–750 kcal/day, and the daily caloric content should be 1000–1200 kcal/day for women and 1200–1600 kcal/day for men [61]. The most important aspect of the diet is to establish a constant, yet not excessive, energy deficit [47].

It is crucial to cut down on products with a proven negative impact on health (e.g., foods rich in saturated and trans-unsaturated fats) and replace them with meals providing health benefits. The diet should be adapted to the patient’s needs to ensure the relevant energy deficit and the right proportions between carbohydrates, fats, and proteins, but should also not prompt malnutrition or nutritional deficiencies. In most cases, the recommended proportions of macronutrients (by their caloric content) are as follows: 40–45% carbohydrates, 30–40% fats, and 20–30% proteins [61].

The strongest evidence for beneficial effects on overall health was obtained for the Mediterranean diet [62,63]. Nonetheless, the DASH diet (Dietary Approaches to Stop Hypertension) and the low-carbohydrate diet are also beneficial [64,65]. A vegetarian diet, although proven to lower HbA1c, does not lower fasting glycemia [66].

After accomplishing short-term weight loss goals, a support system should be implemented to consolidate the achieved effects and to strengthen them. Maintaining body weight should be a priority over further weight loss [21].

If the patient expresses a strong motivation for both rapid weight loss and maintaining this weight afterward, a very-low-calorie diet (i.e., ≤800 kcal/day) may be considered. It allows for achieving a much greater weight loss (even as much as 15% from the baseline). This option can only be employed by a limited number of patients for up to 3 months (ADA) or 5 months (EASD). The therapy should be carried out under the supervision of a qualified specialist [14,19]. After completing this intensive dietary regimen, patients are more prone to regain their previous body weight than patients undergoing a typical weight reduction program of gradual weight loss [67]. The optimal rate of weight loss is stated to be in the range of 0.5–1 kg every 1–2 weeks [61].

A low-calorie meal replacement regimen (800–850 kcal/day) using a formula diet, shakes, prepackaged meals, etc. can be prescribed as an alternative to a typical MNT [19]. A meal replacement diet should be overseen by a registered dietitian [61,68].

Armstrong et al. argue that a reduction in sweetened drink consumption, while maintaining adequate hydration, also plays a crucial role in the process of weight reduction. Even beverages sweetened with artificial sweeteners can have a negative impact on body weight through the dysregulation of intestinal microbiota [69]. Adequate hydration supports weight loss because water is indispensable for metabolic and biochemical reactions and is lost as a result of perspiration and respiration during strenuous physical exercise.

Alcohol has virtually no nutritional value yet has a high energy content; therefore, alcohol consumption during obesity treatment should be kept to a bare minimum [61].

## 6. Physical Activity in Obesity Therapy in T2DM

Exercise, both aerobic and anaerobic, improves diabetes control, with greater benefits produced by aerobic exercise [70]. The major advantage of resistance training, on the other hand, comes from the fact that it can be safely performed by patients who must refrain from aerobic activity [21].

On average, physical activity allows for a decrease in HbA1c value by 0.6% [71]. Aerobic physical activity should be undertaken daily and should last for ≥30 min/day and ≥150 min/week [51,72]. The greatest weight reduction is achieved through everyday physical activity lasting 60–90 min/day [61]. Resistance training should be performed at least twice a week, or even 3–4 times a week [61,72].

Physical activity alone results in less weight loss than diet alone (2.0 kg vs. 4.0 kg) [72,73,74]. However, routine physical activity, even if it not leading to significant weight loss, brings general health benefits such as a reduction in cardiovascular risk, a decrease in arterial blood pressure, an improvement in insulin sensitivity and dyslipidemia, improved mood, and an improvement in QoL [21,75,76]. This is principally due to the increase in lean body mass (muscles, bones) [73]. Physical activity protects from the loss of muscle mass while being on low-caloric MNT regimens. It also prevents certain types of cancer [77]. All these benefits translate into lower all-cause mortality [77].

More frequent but shorter training sessions bring about more benefits compared to longer yet fewer exercise sessions [78].

A comparison of the net effects of various forms of physical activity (aerobic exercise vs. resistance exercise) on weight reduction remains a controversial topic, as noted by Yang et al. [79]. Most guidelines assume that aerobic activity results in greater weight reduction by strictly reducing body fat mass. Resistance exercise, on the other hand, promotes lean body mass gain, or more specifically, muscle mass gain or at least slowing muscle mass loss [72]. This is because a vast majority of adipose tissue reduction cases lead to a concomitant decrease in muscle mass. Thus, even putting a halt to the decline in muscle mass results in a relative increase in muscle mass proportion compared to fat mass. Boulé et al. argue that the greatest effectiveness of weight reduction is achieved by combining both types of physical activity, i.e., aerobic activity and resistance training since both types of training will reduce insulin resistance. The synergistic effect of these two types of physical activity is due to the different mechanisms of action. Aerobic training leads to a decrease in insulin resistance by reducing visceral fat mass, while resistance exercise contributes to an increase in insulin sensitivity through increased glucose expenditure by hypertrophied muscles [72].

The physical training regimen should be adjusted to the patient’s health status and discussed with an exercise physiologist [61]. Before the program is initiated, potential contraindications should be taken into account (cardiovascular diseases, proliferative retinopathy, etc.). Recommended activities include walking, jogging, and swimming, as well as some hobbies (e.g., gardening).

After achieving weight goals, the ADA recommends 200–300 min of activity per week during the phase of weight maintenance [14].

In addition to being involved in conventional physical activity, it is strongly advised to limit a sedentary lifestyle, i.e., by taking a brief walk for 2 min after every 30 min of work in a sitting position [80].

## 7. Obesity Pharmacotherapy and Antihyperglycemic Treatment

### 7.1. Antihyperglycemic Agents and Their Impact on Body Weight

The effect of antihyperglycemic agents on body weight should be taken into account in patients with obesity and T2DM. If possible, diabetes treatment should be conducted with medications facilitating weight loss. In particular, GLP1-RAs (glucagon-like peptide-1 receptor agonists) and SGLT2is (sodium/glucose cotransporter 2 inhibitors) should be favored [19,81].

At the time of drug selection, costs of therapy, patient preferences and, above all, comorbidities should be given due concern. For instance, GLP1-RAs and SGLT2is are beneficial to patients with cardiovascular diseases [81]. If the use of antihyperglycemic drugs with weight-reducing effects is not feasible for some reasons (e.g., for the sake of their high cost), drugs with a neutral effect on body weight should be utilized [14]. These agents include metformin, DPP4 inhibitors, and α-glucosidase inhibitors [17,19].

If the patient’s state necessitates insulin therapy (the antihyperglycemic drug with the greatest potential for weight gain), it is recommended to use a combination drug containing long-acting insulin and GLP1-RA instead, such as degludec/liraglutide or glargine/lixisenatide. GLP1-RA counteracts the increase in body weight due to insulin action, and the overall effect of the combination drug remains neutral to body weight [19].

Among the drugs used to treat T2DM is liraglutide, and in some countries semaglutide has also been officially approved for the treatment of obesity. It should be noted, however, that the dosage of GLP1-RAs in weight management is different than in T2DM management [14,19,82].

A comparison of antihyperglycemic drugs, including their effect on body weight, is presented in Table 2.

### 7.2. Antihyperglycemic Agents Facilitating Body Weight Reduction

#### 7.2.1. SGLT2is

SGLT2 inhibitors reduce the reabsorption of glucose in the kidneys, and, by virtue of that, glucose is excreted into the urine [84]. Since the action of SGLT2is is dependent on adequate renal function, eGFR (estimated glomerular filtration rate) should be monitored periodically in accordance with FDA (United States Food and Drug Administration) and EMA (European Medicines Agency) recommendations before commencement and during therapy. All SGLT2is promote weight reduction by about 2.0–3.0 kg, regardless of the specific drug used (drug class effect).

SGLT2is are used in diabetology, e.g., SGLT2is are indicated in heart failure regardless of ejection fraction (reduced or preserved) and in adults with chronic kidney disease [85,86,87,88]. Beneficial effects on the cardiovascular system (e.g., in heart failure) have been proven for empagliflozin, canagliflozin, and dapagliflozin. The most important side effect of SGLT2is is the increase in genitourinary tract infection risk due to glucose excretion, a medium for microorganism growth [89,90].

#### 7.2.2. GLP-1RAs

All formulations of GLP-1-RAs are administered mostly by subcutaneous injection (with an exception for semaglutide, which is available in both subcutaneous and oral formulations) [91,92]. GLP-1RAs induce a glucose-dependent stimulation of insulin secretion and inhibition of glucagon secretion. Additionally, GLP-1Ras exert a feeling of satiety by affecting POMC/CART (pro-opiomelanocortin/cocaine- and amphetamine-regulated transcript) neurons in the CNS (central nervous system) and delaying postprandial gastric emptying. These actions in tandem promote weight reduction [82,93,94,95]. GLP-1RAs can be readily subdivided into two groups: (1) short-acting, administered at least once a day (exenatide, lixisenatide, liraglutide) and (2) long-acting, administered once a week (dulaglutide, exenatide extended-release, semaglutide). Research so far indicates that glucose is most effectively lowered by (in order of efficacy) semaglutide, dulaglutide, and liraglutide [96,97,98]. The weight loss effect is representative of the entire drug class. Liraglutide, semaglutide, and dulaglutide also exhibit beneficial cardiovascular effects [99,100,101]. The most common adverse effects are gastrointestinal ailments, e.g., nausea, vomiting, and diarrhea. These are most pronounced at the very beginning of therapy and decrease or disappear altogether over time. Contrary to the early conjectures, the latest studies appear not to corroborate the suspicions of the increase in acute pancreatitis or pancreatic cancer risk due to the GLP-1RAs [102]. Nevertheless, there are hints that GLP-1RAs may precipitate gallbladder diseases [95,102,103].

According to official product characteristics for liraglutide issued to the EMA (European Medicines Agency), the only contraindication for liraglutide use is hypersensitivity to the active substance or any of the excipients contained within the drug [104]. Possible side effects are not yet fully corroborated and remain under investigation. These potential side effects, enumerated in the aforementioned product characteristics, include pancreatitis, cholelithiasis and cholecystitis, thyroid disease (e.g., goiter, medullary thyroid carcinoma) [105,106,107,108], increased heart rate, dehydration, and—although rare compared to other antidiabetic agents—hypoglycemia. Moreover, the safety of liraglutide has not been established in patients aged 75 years or more, patients with severe renal or hepatic impairment, patients treated with other products for weight management, and patients with obesity secondary to endocrinological or eating disorders [104]. Liraglutide is registered for use in patients aged 10 years or more (in case of T2DM) and aged 12 years or more (in case of obesity) [104].

The same precautions hold mostly true for semaglutide formulations with the exception of cholelithiasis and cholecystitis, as well as thyroid disease, which is not reported in the official product characteristics for semaglutide [109]. However, there is a warning of potential diabetic retinopathy associated with semaglutide use, possibly due to a risk of achieving rapid glucose normalization [109].

GLP-1RAs are contraindicated in women of childbearing potential due to a lack of safety data during pregnancy and breastfeeding [104,109].

As mentioned earlier, liraglutide and semaglutide are the only GLP-1RAs (and the only antihyperglycemic agents for that matter) approved for weight management in patients without concomitant T2DM [82,110]. 

## 8. Weight Loss Medications

The drugs used in the treatment of obesity are intended for patients with (1) BMI ≥ 30 kg/m^2^ or (2) BMI ≥ 27 kg/m^2^ and accompanying disease(s) resulting from overweight/obesity [14]. It should be recognized that medications used in the treatment of obesity are only an adjunct to the main therapy and cannot replace other methods of obesity management. Prior to the commencement of pharmacotherapy and throughout its duration, the patient must display a strong motivation to reduce body weight through the use of proper diet and physical activity. Table 3 provides detailed information on the effects of weight loss medication.

If the patient does not achieve significant weight loss after 3 months of pharmacotherapy (≥5% weight reduction compared to the baseline), the drug should be discontinued. The same applies if the patient has experienced significant side effects or if the patient does not conform to the physician’s recommendations regarding drug use [14].

Most drugs used in the treatment of obesity are intended for long-term use. Phentermine, discussed later in this article, is an exception and is licensed for short-term use only [115].

**Figure 2:**
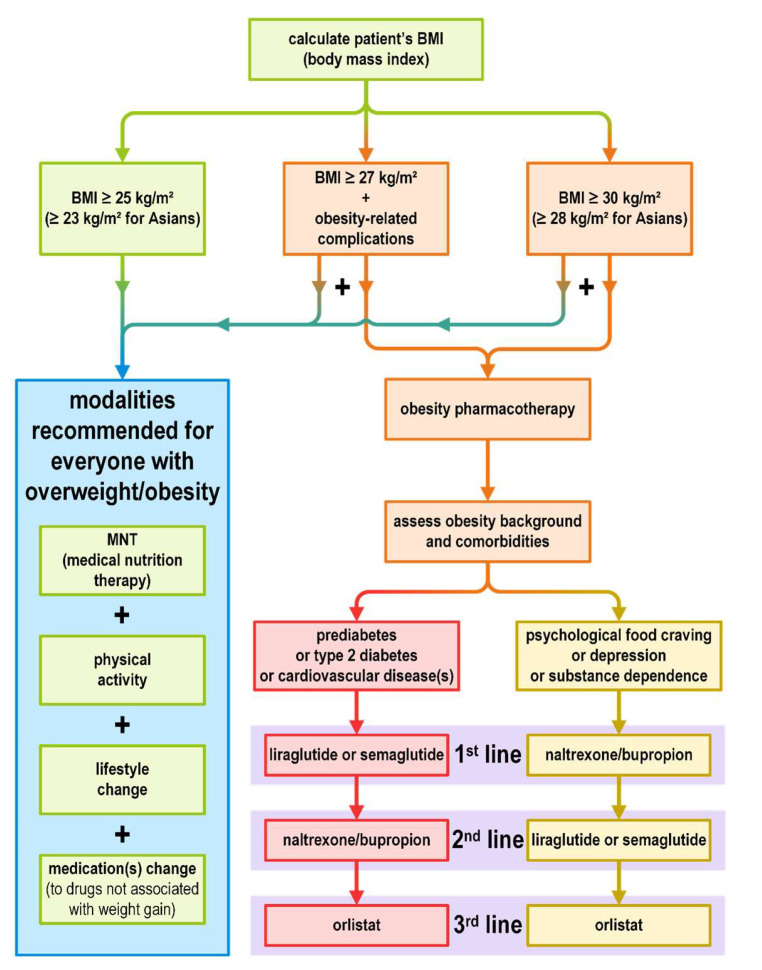
Overweight and obesity management algorithm (based on various recommendations) [20,21,81,95].

The proposed algorithm of pharmacotherapy selection depending on obesity background is shown in Figure 2 (an amalgam based on various recommendations).

As was stated previously in relation to GLP-1Ras, all weight-reducing agents are contraindicated in women of childbearing potential due to a lack of safety data during pregnancy and breastfeeding [104,109,116,117]. Therefore, contraceptive use is strongly indicated in all women of the aforementioned populations taking these medications [116].

### 8.1. Liraglutide and Semaglutide

Liraglutide and semaglutide were discussed (in conjunction with other drugs in its class) in the GLP1-RAs section.

Here, some additional benefits of GLP1-RAs in weight reduction therapy will be emphasized. As evidenced by the data presented in Table 3, GLP-1RAs offer the greatest benefit in the treatment of patients with type 2 diabetes co-occurring with obesity [14,20,95]. This is because they are among the most effective weight loss medications. Additionally, GLP-1RAs provide numerous other benefits in the treatment of T2DM. They improve the metabolic control of diabetes (e.g., they lead to a decrease in glycated hemoglobin and improvement in glycemic control in ambulatory measurements), they lower blood pressure, and they have nephroprotective and cardioprotective capabilities. This all translates to a reduction in cardiovascular risk (a decrease in the risk of stroke and myocardial infarction), and consequently a longer lifespan with a higher quality of life.

Furthermore, in patients with obesity but not carbohydrate abnormalities, GLP-1RAs exhibit the strongest protective effect against the development of overt T2DM.

Another advantage of GLP1-RAs is their safety due to the glucose-dependent mechanism of action resulting in reduced risk of hypoglycemia. Compared to other drugs used in the treatment of obesity, GLP1-RAs have far fewer contraindications and side effects. As was already stated in Section 7.2.2., a significant number of side effects are still under investigation and have not yet been fully confirmed.

### 8.2. Orlistat

Orlistat, a strong, selective pancreatic and gastric lipase inhibitor, blocks the hydrolysis of TGs contained in consumed food. Therefore, approximately 30% of TGs are not digested into FFAs (free fatty acids) and are excreted in feces [118]. It is the only weight management drug that does not affect the hypothalamic satiety nuclei in any way. Studies show that orlistat reduces the risk of prediabetes to T2DM progression [119,120]. The most common adverse effects result directly from the drug’s mechanisms of action, which are fatty stools, diarrhea/steatorrhea, and flatulence [95]. Cases of rectal bleeding have also been reported [116]. Orlistat affects the absorption of many drugs, including levothyroxine, cyclosporine, and anticonvulsants. Therefore, the co-administration of orlistat with other drugs may lead to potential interactions. The use of these drugs should be closely monitored if possible (e.g., levothyroxine). If not feasible, a substitute that is not subject to interaction should be used. If this is also impossible, the use of orlistat should be discontinued altogether, especially in cases when the other drug is prioritized over orlistat [116].

Contraindications for orlistat use include hypersensitivity to the active substance or any of the excipients, chronic malabsorption syndrome, and cholestasis. The use of orlistat in women of childbearing potential is also contraindicated (during pregnancy or breastfeeding). Due to potential malabsorption, an additional contraceptive method is advised in addition to oral contraceptives to prevent potential pregnancy [116].

There were cases of nephropathy due to hyperoxaluria associated with orlistat use, especially in patients with concomitant chronic kidney disease or dehydration. Hypothyroidism was also described, possibly because of decreased dietary iodine absorption [116].

In order to avoid a deficiency of fat-soluble vitamins (A, D, E, K), their supplementation is recommended during orlistat therapy [121]. Moreover, patients taking oral anticoagulant INR (international normalized ratio) values, which are dependent on vitamin K concentrations, should be carefully monitored. Orlistat can be used only in adult patients [116].

### 8.3. Naltrexone/Bupropion

Naltrexone/bupropion is a combination drug. Naltrexone, an opioid receptor antagonist, has been used for many years in the therapy of addictions (alcohol and opioid dependence). Bupropion, an antidepressant belonging to NDRIs (norepinephrine and dopamine reuptake inhibitors), much like naltrexone, is used to treat addiction (tobacco dependence).

Bupropion increases the synthesis and secretion of α-MSH (α-melanocyte stimulating hormone) and β-endorphin from POMC cells in the ARC (arcuate nucleus) of the hypothalamus and, thus, induces a feeling of satiety. A naltrexone blockade of the β-endorphin inhibitory effect on POMC cells enhances the orexigenic effect provided by bupropion [122]. By affecting the reward system, naltrexone/bupropion further inhibit appetite [123].

There are many contraindications for naltrexone/bupropion use, as listed in the product characteristics [117]. These include hypersensitivity to the active substance(s) or any of the excipients; uncontrolled hypertension; current seizure disorder or a history of seizures; central nervous system tumor; acute alcohol or benzodiazepine withdrawal; bipolar disorder; concomitant treatment with other drugs containing bupropion or naltrexone; current or previous diagnosis of eating disorders (bulimia nervosa, anorexia nervosa); opioid dependence or acute opiate withdrawal; concomitant use of MAOIs (monoamine oxidase inhibitors); and lastly, severe renal or hepatic impairment [117]. Moreover, naltrexone/bupropion use was reported to increase suicidal behaviors in some patients with psychiatric disorders. There is suspicion that the drug lowers the seizure threshold, which may potentially result in seizures. The contraindication to the naltrexone/bupropion use in patients with uncontrolled blood pressure is due to a minuscule increase in systolic and diastolic blood pressure of 1 mmHg on average during therapy.

The most common side effects of naltrexone/bupropion include nausea, constipation, and headaches [95]. In general, the adverse effects of naltrexone/bupropion are temporary. Naltrexone/bupropion, due to its strong effect on hepatic metabolism, induces interactions with numerous other drugs, such as beta-blockers, SSRIs (selective serotonin reuptake inhibitors), antipsychotics, TCAs (tricyclic antidepressants), some antiarrhythmics, antiplatelet drugs, antiviral drugs, etc. [124]. Potential interactions between naltrexone/bupropion and drugs already taken by the patient should be accounted for before starting naltrexone/bupropion treatment.

### 8.4. Phentermine

Phentermine is a sympathomimetic approved solely for short-term treatment of obesity (≤12 weeks). Phentermine acts in the CNS by increasing the release of norepinephrine, and—to a lesser extent—by increasing the release of serotonin and dopamine [125]. By affecting the hypothalamic nuclei, phentermine reduces hunger, and by affecting adipocytes, stimulates lipolysis. Adverse effects include insomnia, dry mouth, constipation, and dizziness [20]. An undisputed advantage of phentermine is its low cost (it is the cheapest drug approved for weight management) [14,82].

### 8.5. Phentermine/Topiramate

This is an orally taken combination drug. Phentermine has been discussed in the previous section. Topiramate is an anticonvulsant drug that works by inhibiting carbonic anhydrase and amine/GABA (gamma-aminobutyric acid) neurotransmission modulation. Most likely, topiramate inhibits the hunger at the CNS level while increasing energy expenditure at the same time [121,126]. Research suggests that phentermine/topiramate has a beneficial effect on TC (total cholesterol) and LDL (low-density lipoprotein) concentrations. Adverse effects of phentermine/topiramate are generally similar to phentermine [121].

## 9. Medications for Comorbidities and Their Impact on Body Weight

Patients with T2DM and obesity commonly have numerous other comorbidities. Some of them are direct/indirect complications of diabetes and obesity, and some of them are independent of diabetes and obesity. Due to multimorbidity and polypharmacy in this group of patients, a physician attempting obesity therapy should analyze the list of medications taken by the patient because many drugs favor weight gain. A large proportion of obesity-promoting medications act on the CNS. These are atypical antipsychotics (olanzapine, clozapine, quetiapine, risperidone, aripiprazole), some typical antipsychotics (haloperidol), anti-depressants (amitriptyline, imipramine, nortriptyline, mirtazapine, MAOIs (monoamine oxidase inhibitors)), anticonvulsants (gabapentin, carbamazepine, valproic acid), and antihistamines [20,127,128].

A number of medications for somatic diseases can also have weight-gain effects, e.g., beta-blockers and protease inhibitors. Many commonly used hormonal drugs, i.e., GCSs (glucocorticoids) and some OCs (oral contraceptives), promote obesity [128].

On the other hand, some medications may be beneficial for obesity. Drugs with a supportive effect on weight management include fluoxetine, duloxetine, sertraline, venlafaxine, and bupropion (used in combination with naltrexone in the pharmacotherapy of obesity). Usually, however, evidence of their weight-loss effect is weak, incomplete, or of limited value [127,129,130].

After reviewing the list of medications taken by the patient, the physician should switch the patient to drugs with a neutral effect on body weight, or preferably to medications with an additional weight-loss effect. The drug replacement should take place with consent from the specialists treating the underlying diseases [14].

## 10. Conclusions

Obesity is a disease with a complex etiology and a significant impact on patients’ health. It causes numerous comorbidities and complications, of which T2DM is one of the most common, and cardiovascular complications are the gravest ones. Due to the significant costs of obesity-related complications therapy, it is paramount to manage obesity effectively in order to avoid negative ramifications.

Obesity and T2DM are intertwined in a pathogenetic vicious cycle. Therefore, an efficient treatment of obesity leads to improvement of diabetes control and, conversely, T2DM management with appropriate drugs increases the chance of achieving weight reduction.

Bearing all this in mind, obesity treatment should be carried out in a professional setting to ensure the greatest likelihood of success. Weight management should be supervised by a qualified, multi-disciplinary team providing the patient with full access to necessary information and individually tailored therapy regimens. No single method can provide optimal results. A desirable outcome is attainable by a combination of diverse methods such as diet therapy, lifestyle change, and physical activity. Pharmacotherapy of obesity or bariatric surgery may be instrumental, but only as auxiliary methods. A custom-tailored approach is able to bring about tangible results such as an improvement in QoL and comorbidities, a reduction in all-cause mortality, and a reduction in medication dosage.

## Figures and Tables

**Table 1:** Classification of nutritional status according to BMI value and WC.

Classification		BMI Value (kg/m^2^)		WC (cm)		Risk of Developing Body Mass Related Diseases
		Non-Asian	Asian	Non-Asian	Asian	
underweight		<18.5	<17.5	nd *	nd *	↑
normal weight		18.5–24.9	17.5–22.9	nd *	nd *	N
overweight		25.0–29.9	23.0–27.9	nd *	nd *	↑
obesity	class I	30.0–34.9	28.0–32.4	♀ > 88 * ♂ > 102 *	♀ > 80 * ♂ > 90 *	↑↑
	class I	35.0–39.9	32.5–37.4	nd *	nd *	↑↑↑
	class III	≥40.0	≥37.5	nd *	nd *	↑↑↑↑

BMI—body mass index; WC—waist circumference; N—normal risk; ↑—higher risk. nd—no data. * Waist circumference measurement is only applicable for central obesity diagnosis and cannot be used for diagnosis of other body weight abnormalities.

**Table 2:** Antihyperglycemic agents and their effect on weight [16,17,19,81,83].

Drug Class	Mechanism of Action	Drug Sub-Class		Drugs	Weight Change	HbA1c Decrease [mmol/mol]	Cost
**biguanide**	↑ insulin sensitivity ↓ hepatic glucose production			metformin (conventional or extended-release)	NC	10.9	$
**incretin**	↑ satiety (only GLP1-RA), ↑ glucose-dependent insulin secretion ↓ stomach emptying ↓ glucagon secretion		**GLP1 receptor** **agonist**	short-acting: exenatide, lixisenatide, liraglutide ^†^ long-acting: dulaglutide, exenatide extended-release, semaglutide ^†^	↓ 1.1–4.4 kg	6.6–15.3	$$$$
			**DPP4 inhibitor**	sitagliptin, vildagliptin, saxagliptin, linagliptin, alogliptin	NC	5.5–7.6	$$$
**SGLT2 inhibitor**	↓ renal glucose resorption			canagliflozin, dapagliflozin, empagliflozin, ertugliflozin	↓ 2.0–3.0 kg	5.5–7.6	$$$
**α-glucosidase inhibitor**	↓ carbohydrate digestion and absorption (↓ action of α-glucosidase and α-amylase)			acarbose, miglitol	NC	7.6–8.7	$$
**insulin**	direct substitution of endogenous insulin action	**rapid-acting**		aspart (conventional and fast-acting), glulisine, lispro	↑ *	≥ 9.8 *	$–$$$$ *
		**intermediate-acting**		human NPH			
		**long-acting**		degludec, detemir, glargine			
**insulin +** **GLP1-RA**	(see GLP1-RA and insulin sections)			degludec/liraglutide, glargine/lixisenatide	NC	ND	$$$$
**insulin secretagogue**	↑ insulin secretion (glucose-independent)	**sulfonylurea**		glibenclamide (glyburide), glipizide, gliclazide, glimepiride	↑ 1.2–3.2 kg	6.6–13.1	$
		**meglitinide**		repaglinide, nateglinide	↑ 1.4–3.3 kg	7.6–12.0	$$
**TZD**	↑ insulin sensitivity (activation of PPAR-γ receptors)			pioglitazone, rosiglitazone	↑ 2.0–2.5 kg	7.6–9.8	$$$

NPH—neutral protamine Hagedorn; PPAR-γ—peroxisome proliferator-activated receptor-gamma; TZD—thiazolidinedione; NC—no change; ND—no data; beneficial effect on body weight highlighted in green; neutral effect on body weight highlighted in yellow; adverse effect on body weight highlighted in red. ^†^ Liraglutide and semaglutide are the only antihyperglycemic drugs approved for the pharmacotherapy of obesity; * drug- and dose-dependent. The number of $ symbols correspond to the cost of the drugs; ↑ = an increase, ↓ = a decrease.

**Table 3:** Drugs used in the pharmacotherapy of obesity [14,95].

			Orlistat	Liraglutide, Semaglutide ^†^	Naltrexone/Bupropion	Phentermine/Topiramate	Phentermine ^‡^
**mechanism of action** [20]			gastric/pancreatic lipase inhibitor	GLP1 receptor agonist	opioid receptor antagonist + NDRI	sympathomimetic + CAI and GABA/amine modulator	sympathomimetic
**route of administration**			oral	subcutaneous	oral	oral	oral
**target dose**			120 mg	3.0 mg (lirglutide) 2.4 mg (semaglutide)	16 mg/180 mg	15 mg/92 mg	15 mg
**dosage frequency**			three times daily	once daily (liraglutide)once weekly (semaglutide)	twice daily	once daily	once daily
**long-term weight change vs. placebo**		**%**	−4.0% [14], −2.9% [95]	liraglutide: −4.0% [14], −5.4% [95], from −6.0% to −8.0% [20] semaglutide: −15.8% * [111]	−4.2% [14], −4.8% [95], from −8.2% to −11.5% [20]	−8.6% [14], −7.3% [112], −9.4% [113] ^§^, from −5.1% to −10.9% [20]	−4.9% [14] (short-term effect)
		**kg**	−2.8 kg, −3.0 kg [20]	liraglutide: −4.2 kg; −6.8 kg * [111] semaglutide: −15.3 kg * [111]	ND	−6.4 kg [112]	ND
**change in T2DM development risk**			↓ (−37.3%)	↓ (−79.0%)	ND	ND	ND
**effect on HbA1c [mmol/mol]**			−4.4	−10.9	−5.5	ND	NC/NS [114]
**effect on lipids**	**TC**		↓	↓	ND	↓ [112,113]	NC/NS [114]
	**LDL**		↓	↓	↓	↓ [112,113]	NC/NS [114]
	**non-HDL**		ND	↓	ND	ND	ND
	**HDL**		NC/NS	↑	↑	NC/NS [112]/↑ [113]	NC/NS [114]
	**TG**		NC/NS	↓	↓	NC/NS [112]/↓ [113]	NC/NS [114]
**cost of therapy (per month)**			$$	$$$$	$$$	$$	$

T2DM—diabetes mellitus type 2; NDRI—norepinephrine and dopamine reuptake inhibitor; CAI—carbonic anhydrase inhibitor; ND—no data; NC/NS—no change or not significant; TC—total cholesterol; LDL = low-density lipoprotein; HDL—high-density lipoprotein; TG—triglyceride; beneficial effect highlighted in green; non-conclusive data on beneficial effect highlighted in yellow. Lorcaserin and lorcaserin SR were not included in the comparison due to drug approval being withdrawn by the FDA as of 13 February 2020 [15]. All data are based on [14,95], unless otherwise stated. ^†^ Liraglutide and semaglutide are the only drugs currently approved for therapy of both diabetes mellitus type 2 and obesity. ^‡^ Alone, phentermine is approved only for short-term obesity management (≤12 weeks). * Not adjusted for placebo [111]. ^§^ Based on analysis A (prespecified ITT-LOCF—Intent to Treat population Using the Last Observation Carried Forward Method). ↑ = an increase, ↓ = a decrease. The number of $ symbols correspond to the cost of the drugs.

## Data Availability

Not applicable.
